# Toxicity Evaluation of Pũrṇa Cantirotaya Centũram, a Siddha Medicine in Wistar Rats

**DOI:** 10.1155/2015/473296

**Published:** 2015-01-26

**Authors:** B. Chitra, R. S. Ramaswamy, V. Suba

**Affiliations:** ^1^National Institute of Siddha, Tambaram Sanatorium, Chennai 600047, India; ^2^Central Council for Research in Siddha, Arumbakkam, Chennai 600106, India

## Abstract

Pũrṇa Cantirotaya Centũram (PCC), a herbometallic formulation of Siddha medicine, consists of mercury, sulphur, and gold, processed with red cotton flower and plantain stem pith juices. To evaluate its safety, acute and 28-day repeated oral toxicity studies were performed following OECD test guidelines 423 and 407, respectively. In acute study, PCC was administered orally at 5, 50, 300, and 2000 mg/kg body weight. Animals were observed for toxic signs for 14 days. Gross pathology was performed at the end of the study. In repeated dose toxicity study, PCC was administered at 2.5, 25, and 50 mg/kg body weight daily for 28 days. Satellite groups (control and high dose) were also maintained to determine the delayed onset toxicity of PCC. In acute toxicity study, no treatment related death or toxic signs were observed. It revealed that the LD_50_ cut-off value of PCC is between 2000 and 5000 mg/kg body weight. The repeated dose study did not show evidence of any treatment related changes in all observations up to the high dose level, when compared with the control. Histopathological examination revealed no abnormalities except mild hyperplasia of stomach in high dose group. This study provides scientific validation for the safety of PCC.

## 1. Introduction

Siddha system of medicine dates back to many centuries. It has been practiced by Tamil vaithiyars (native healers of Tamil Nadu) who taught people an impeccable life style that trails in societies of Tamil Nadu. The current accepted modern medicine has gradually developed over the years by scientific and observational efforts of scientists. However, the basis of its development remains rooted in traditional medicine and therapies [1]. The scientist Paracelsus quoted that all substances are poisons; there is none, which is not a poison. The right dose differentiates a poison from a remedy. Likewise, the liability of inimical effects while administering the metals and minerals containing traditional medicines is to be explored for its safeness. In Siddha system of medicine, the raw materials like plant, mineral, and animal resources are acquired from the natural surroundings. They have been used extensively for many centuries after thorough evaluation of the drug by traditional way. Siddha system emphasizes the dose regimen and pertinent vehicle for every medicine intake. Metals have been used as therapeutics since time immemorial. Siddha medicine has vast range of mercurial usage in therapeutic application for treating acute and chronic diseases [2]. Pũrṇa Cantirotaya Centũram (PCC) is one of the classical formulations in Siddha system of medicine, with wide range of therapeutic uses. Centũram is one of the 32 types of internal medicines in Siddha system of medicine. It is considered to be very potent form of drug having extensive shelf life up to 75 years and it provides speedy recovery from many of the chronic illnesses [3]. Centũram is oxidized product of metals or minerals, which are free from metallic residue of its parent substances. Siddha medicine describes testing of the end products by some simple checks. It insists on the color description, the drug should be fine enough to enter the crevices of finger, and it should float on the water. It ensures the correct attainment of final product. This physical testing qualifies a compound to become a drug entitled for human usage [4]. Pũrṇa Cantirotaya Centũram is prepared with gold, mercury, and sulphur. These are the important metals and mineral in Siddha system of medicine, which contributes many therapeutic formulations. Gold medicine in Siddha system is recommended for tuberculosis, low grade chronic fevers, neuromuscular weakness, bronchial asthma, rheumatoid arthritis, diabetes mellitus, and nervous system diseases and seminal insufficiency. It acts as a rejuvenator that slows down ageing [5]. It is claimed to possess general tonic, hepatotonic, cardiotonic, nervine tonic, aphrodisiac, detoxicant, anti-infective, and anti-aging properties [6]. Navabal Rasayan, an Ayurvedic medicine, is used for multiple sclerosis, which contains iron, arsenic, lead, mercury, and gold in different quantity which was not showing any apparent untoward effect during acute and chronic toxicity studies [7]. Gold is widely used in modern medicine for the treatment of rheumatoid arthritis [8]. Gold containing Ayurvedic preparation Swarn Vasant Malti did not show any toxic effect on human volunteers and seemed to increase sperm motility and prostatic activity [9]. Acute oral administration (continuous for 8 weeks on albino mice; 10 mg/20 g b.w./day) of swarna bhasma had not shown any toxic effects as assessed by liver function tests and histological investigations [10]. The toxicity study of an Ayurvedic drug, Rasa sindoor, a mercurial preparation, reported that it did not have any toxic effect on kidneys and its functioning; hence it is safe in animal models [11]. Toxicity study of Siddha medicine Rasagandhi Mezhugu (RGM) in rats reveals that in acute toxicity study 1.5 g/kg body weight RGM given orally to rats did not induce death, suggesting the safety of the preparation. This concentration is 150 times higher than the therapeutic dose. Similarly, during chronic studies 75 times higher concentration than the therapeutic dose for 60 days did not produce any significant organ or haematological toxicity in animals [12]. There is a need to assure the safety of herbal formulations in order to acquire their maximum benefits even though these have been proven to be efficacious in pharmacological studies or by clinical evaluation [13]. While research on medicinal plants has received considerable attention, the mineral preparations have relatively been neglected. Studies on the role of elements in health and disease have now become of global importance with rise of research activity in the last two decades [14–16]. The present study was undertaken to assess the safety of Pũrṇa Cantirotaya Centũram in Wistar rats.

## 2. Materials and Methods

### 2.1. Procurement and Authentication of Raw Drugs

The raw drugs of gold, mercury, and sulphur were purchased from local market in Chennai. They were authenticated by Research Officer (Chemistry), Siddha Central Research Institute, Arumbakkam, Chennai. The red cotton flower (*Gossypium arboreum*) and plantain stem pith juices are used in the preparation process. They were collected from Herbal Garden, National Institute of Siddha, Chennai. These plants were authenticated by Assistant Professor (Botany), National Institute of Siddha, Chennai. Voucher specimen (NIS/MB/32/2012) was submitted in Laboratory of Medicinal Botany, National Institute of Siddha, Chennai.

### 2.2. Preparation of Pũrṇa Cantirotaya Centũram

The raw ingredients were purified as mentioned in Siddha literature [4, 5]. Pũrṇa Cantirotaya Centũram was prepared using the procedure described in Siddha literature [17]. The drug dose is 65–130 mg (i.e.,* 1/2-1 kunri edai*) and can be administered orally, twice daily with honey [17, 18].

### 2.3. Chemicals and Reagents

Surgical spirit was obtained from Kakatiya Pharma, Hyderabad. Formaldehyde and distilled water were obtained from Nice Chemicals, China, and Stangen Fine Chemicals, Hyderabad, respectively. All chemicals were used without further purification. Clinical diagnostic kits were purchased from M/s. Accurex Biomedical Pvt. Ltd., India. All other chemicals and reagents used were of analytical grade.

### 2.4. Animal Care and Husbandry

The study protocol involving animals was reviewed and approved by Institutional Animal Ethical Committee (IAEC), National Institute of Siddha, Chennai, India, with the experimental protocol number NIS/IAEC/I/2011/8, dated June 14, 2011, prior to the initiation of the study. Experiments were performed as per the instructions prescribed by the Committee for the Purpose of Conduct and Supervisions of Experiments on Animals (CPCSEA), Ministry of Environment and Forest, Government of India. Male and female Wistar albino rats, (130–160 g) obtained from National Institute of Nutrition, Hyderabad, were housed in the animal house of National Institute of Siddha, Chennai. Each group of rats was separately housed in polypropylene cages in a ventilated room (air cycles: 15/min; 70 : 30 exchange ratio) under an ambient temperature of 22 ± 2°C and 40–65% relative humidity with 12 hr dark/12 hr light photoperiod. They were provided with food (Nutrilab Rodent feed, Provimi Animal Nutrition India Pvt. Ltd., Bangalore) and purified water* ad libitum*. All the animals were acclimatized to the laboratory conditions at least for 7 days prior to experimentation.

### 2.5. Acute Oral Toxicity Study

The acute oral toxicity study was performed in accordance with Organization for Economic Cooperation and Development (OECD) test guideline 423. It is the principle of the test that is based on stepwise procedure with the each step using three animals. Young, healthy, nulliparous, and nonpregnant Wistar albino female rats were randomly selected for each dose level. They were treated with the starting dose level of 5 mg/kg body weight as a single oral dose of Pũrṇa Cantirotaya Centũram with honey as a vehicle for 3 rats. For control group, honey alone was given to three rats as a single dose at a dose volume of 1 mL/100 g b.w. For oral administration of test drug in rats, PCC was mixed with honey (Dabur India) with the help of mortar and pestle to obtain uniform suspension. After the test drug has been administered, food was withheld for further 3 hours in rats. The animals were observed for morbidity and mortality for first 30 minutes, 1st, 2nd, 4th, and 6th hours on the day of dosing and once a day thereafter up to 14 days. The body weights were recorded individually on 0th, 7th, and 14th day. Then the confirmatory trial using same number of animals is conducted. Following the first step, the next dose levels 50, 300, and 2000 mg/kg body weight were administered in sequence as per the guidelines. The different doses were given to the rats, which were fasted overnight with water* ad libitum*. Observations were made and recorded systematically and continuously after substance administration [19].

### 2.6. Repeated Dose Oral Toxicity Study

A 28-day repeated oral toxicity study was performed according to the OECD test guideline 407. Animals were divided in six groups, each comprising five males and five females. First group was kept as vehicle control whereas the second to fourth groups were administered test drug PCC at the dose of 2.5 mg/kg b.w., low dose (therapeutic dose), 25 mg/kg b.w., mid dose (10xTD), and 50 mg/kg b.w., high dose (20xTD). In order to determine the reversibility or recovery from toxic effects, if any, the satellite groups were preset. Fifth group served as satellite control (received vehicle) and 6th group served as treatment satellite group which received PCC at 50 mg/kg b.w. for a period of 28 days. The satellite groups were scheduled for follow-up observations for the next 14 days without vehicle or PCC administration. The rat dose (2.34 mg/kg b.w.) was derived from the clinical dosage of the drug PCC as given in the literature, by the equivalent surface area dosage conversion from human to rat. The test drug suspensions (PCC with honey) were freshly prepared every day and administered orally (gavage) once daily for 28 consecutive days. Initial body weight of all the groups was recorded. The animals were monitored closely for signs of toxicity throughout the course of study. Appearance and behaviour pattern were assessed daily and any abnormalities in food and water intake were registered [20].

### 2.7. Collection and Analyses of Blood

At the end of the treatment period, the overnight fasted (water allowed) animals were anaesthetized, and blood samples were collected by retroorbital puncture in heparinised (for haematological) and nonheparinised tubes for biochemical analysis (Semi Auto Analyzer, RA 50, Bayer) and serum electrolytes (Electrolyte Analyzer, EC lyte Transasia) [19]. The blood without the anticoagulant was allowed to clot before centrifugation (1610 g at 4°C for 10 min) to obtain serum, which was collected and stored at −20°C until assayed for biochemical parameters the next day. The serum biomarkers analyzed include glucose, triglycerides, cholesterol, SGOT, SGPT, total bilirubin, total proteins, albumin, urea, creatinine, potassium (K^+^), sodium (Na^+^), and chloride (Cl^−^). These tests were assayed using commercial available kits according to the manufacturer's instruction. Blood samples collected in heparinised tubes were used for full blood count (FBC), which included total erythrocytes count (TEC), Hgb, PCV, MCV, MCH, MCHC, platelets count (PLC), and total leucocytes count (TLC). The FBC was analyzed using Automated Hematology System (Transasia) [21].

### 2.8. Histopathology

Necropsy was done in first four groups of animals on day 29, and for the satellite groups it was done on day 42. After collecting blood, the rats were quickly dissected and the organs were removed, freed of fat and connective tissue, blotted with clean tissue paper, and then weighed on a balance. Organs such as brain, lung (right and left), heart, stomach, liver, kidney, thymus, spleen, ovary, and testis were weighed and relative organ weights were calculated. Portions of the tissue from collected organs were used for histopathological examination. Tissues were fixed in 10% neutral buffered formalin (pH 7.2) and dehydrated through a series of ethanol solutions, embedded in paraffin, and routinely processed for histological analysis. Sections of 4-5 *μ*m thickness were cut and stained with haematoxylin and eosin for examination.

### 2.9. Statistical Analysis

The mean changes in body weight, daily food and water intake, organ weight relative to body weight, and biochemical and haematological parameters were statistically analyzed and significant differences within groups were calculated using the one-way ANOVA test followed by Dunnett's test to compare mean differences of control and test drug treated groups. *P* ≤ 0.05 was considered statistically significant. The results were expressed as the mean ± SD. Statistical analysis was performed using the SPSS version 18.

## 3. Results

**Figure 1:**
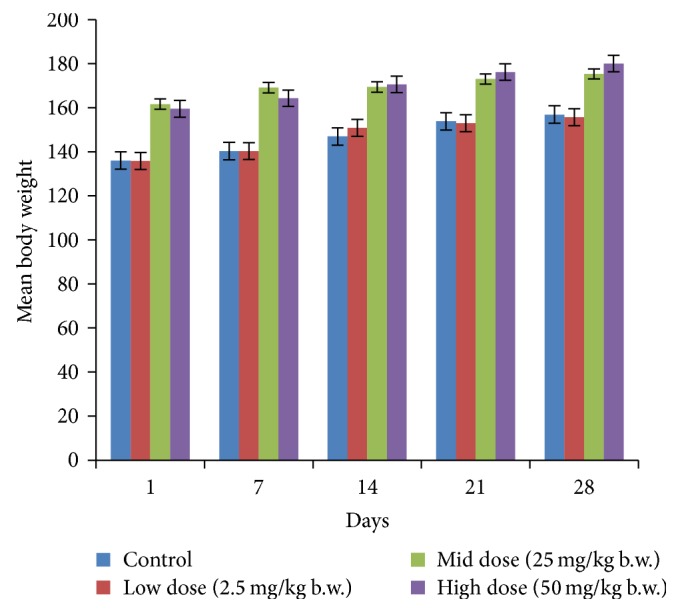
Effect of PCC on body weight of control and PCC treated rats in repeated oral toxicity study. Note: values are expressed as mean ± SD from 10 animals in each group (*P* ≤ 0.05).

**Figure 2:**
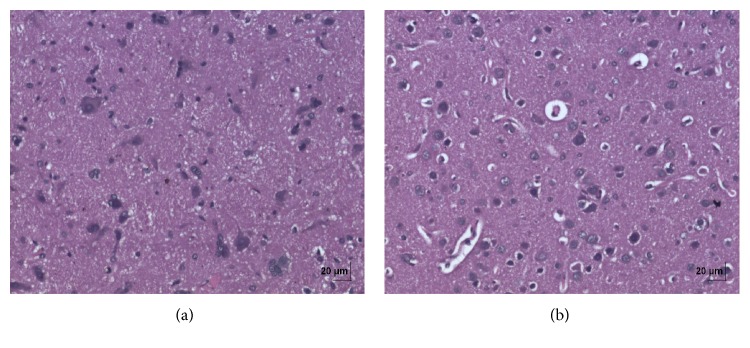
(a) Histopathology of vehicle control brain tissue; organ sections of 4-5 *μ*m thickness were cut and stained with haematoxylin and eosin for histopathological examination. (b) Histopathology of high dose (50 mg/kg b.w.) brain tissue.

**Figure 3:**
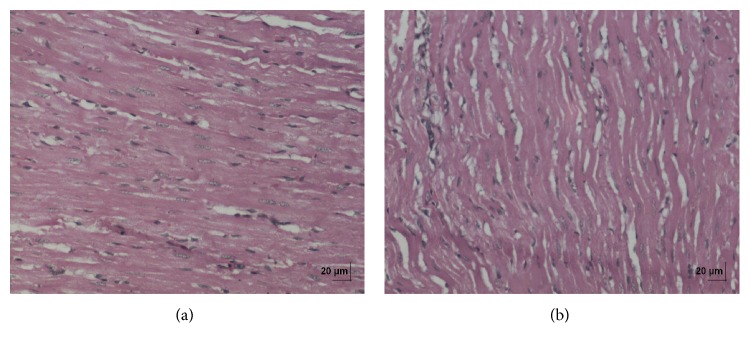
(a) Histopathology of vehicle control heart tissue. (b) Histopathology of high dose (50 mg/kg b.w.) heart tissue.

**Figure 4:**
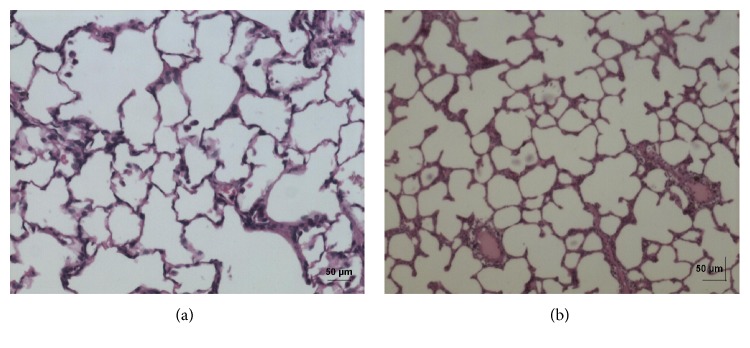
(a) Histopathology of vehicle control lung tissue. (b) Histopathology of high dose (50 mg/kg b.w.) lung tissue.

**Figure 5:**
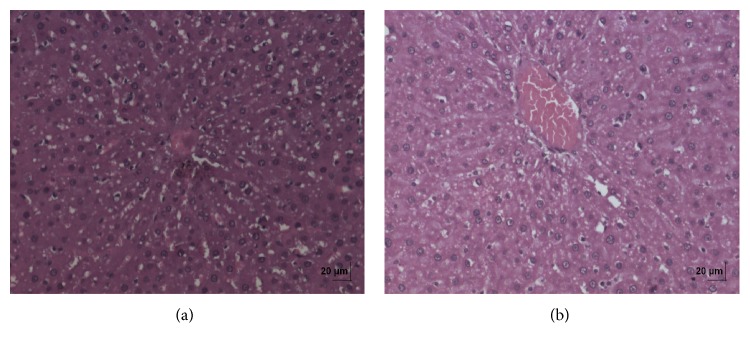
(a) Histopathology of vehicle control liver tissue. (b) Histopathology of high dose (50 mg/kg b.w.) liver tissue.

**Figure 6:**
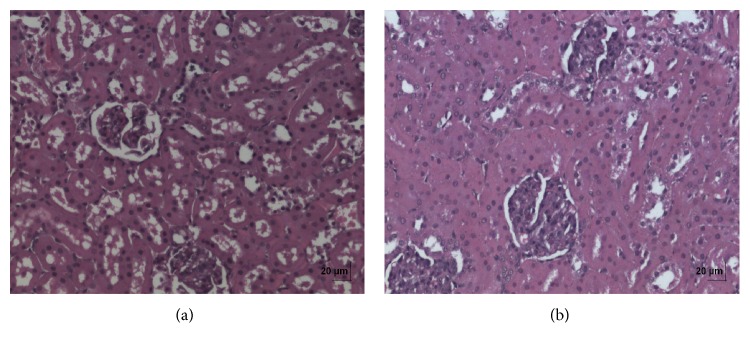
(a) Histopathology of vehicle control kidney tissue. (b) Histopathology of high dose (50 mg/kg b.w.) kidney tissue.

**Figure 7:**
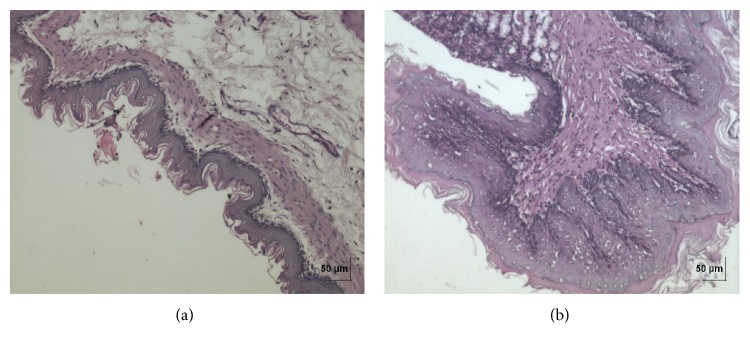
(a) Histopathology of vehicle control stomach nonglandular tissue. (b) Histopathology of high dose (50 mg/kg b.w.) stomach nonglandular tissue showing mild hyperplasia.

**Figure 8:**
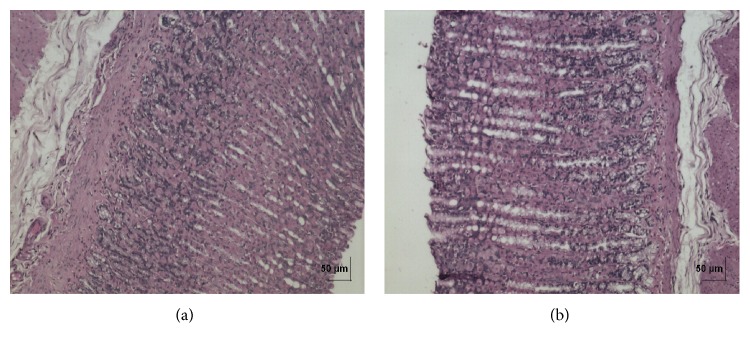
(a) Histopathology of vehicle control stomach tissue glandular part. (b) Histopathology of high dose (50 mg/kg b.w.) stomach tissue glandular part.

**Figure 9:**
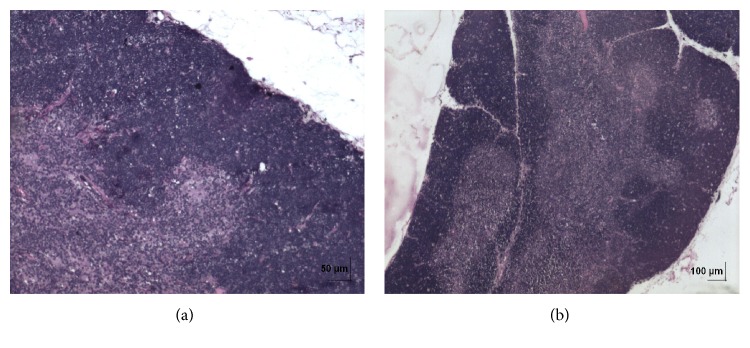
(a) Histopathology of vehicle control thymus tissue. (b) Histopathology of high dose (50 mg/kg b.w.) thymus tissue.

**Figure 10:**
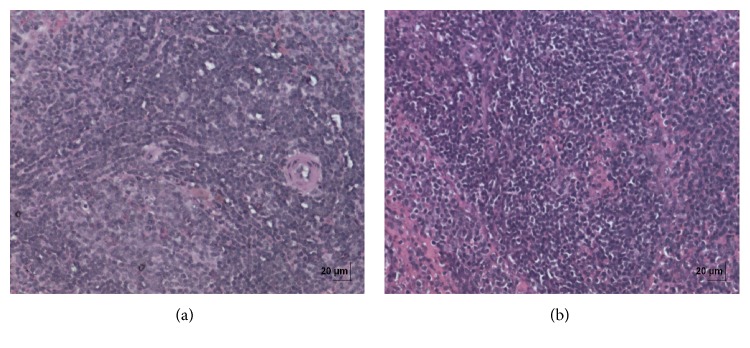
(a) Histopathology of vehicle control spleen tissue. (b) Histopathology of high dose (50 mg/kg b.w.) spleen tissue.

**Figure 11:**
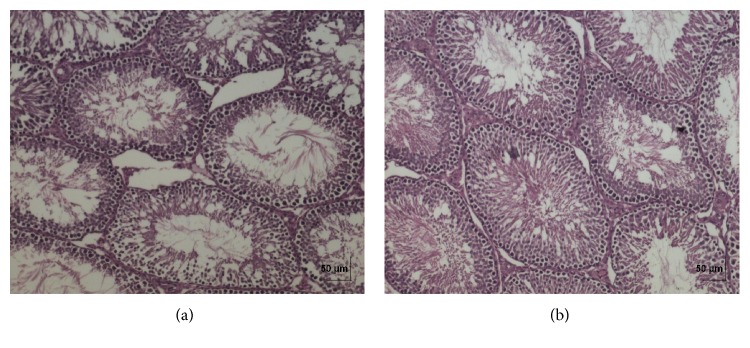
(a) Histopathology of vehicle control testis tissue. (b) Histopathology of high dose (50 mg/kg b.w.) testis tissue.

**Figure 12:**
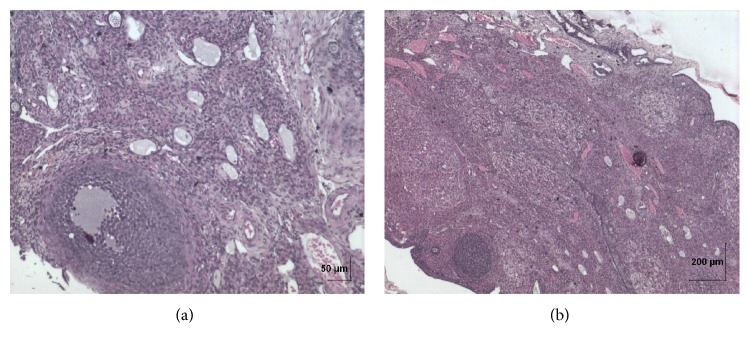
(a) Histopathology of vehicle control ovary tissue. (b) Histopathology of high dose (50 mg/kg b.w.) ovary tissue.

In acute toxicity study, the test drug PCC up to single oral dose of 2000 mg/kg b.w. did not reveal any abnormal clinical signs in any of the animals. All rats survived and no treatment related mortality occurred during the period of 14 days. Gross necropsy did not reveal any abnormal pathology in any of the animals. No significant difference in body weight gain was observed between control and test group rats. Weight loss was not observed in any of the groups treated with PCC. In repeated oral toxicity study all the animals were well oriented and active during the trial period and survived until 28-day treatment period. No signs of clinical toxicity attributable to PCC were observed throughout the study. No changes were observed in food intake and water consumption in the treated groups. No significant (*P* > 0.05) change was observed in the weight of rats after 28 days although there was substantial increase in the weight of rats of both the vehicle control and drug treated groups, but that was not due to effect of drug (Figure 1). There was no statistically significant difference found in hematological parameters (Table 1) between vehicle control and test groups. There were statistically significant differences found in few biochemical parameters such as serum urea, cholesterol, creatinine, and albumin in low dose group (Table 2). The absolute and relative organ weights of both vehicle control and test group rats were found to have nonsignificant differences (Table 3). In histopathological investigations high dose (50 mg/kg b.w.) group exhibits mild hyperplasia in nonglandular area of stomach (Figure 7(b)). There are no pathological alterations found in glandular part of stomach tissue in high dose group (Figure 8(b)). Other organs such as brain, heart, lung, liver, kidney, thymus, spleen, testis, and ovary were revealed to have no abnormalities (Figures 2, 3, 4, 5, 6, 9, 10, 11, and 12).

## 4. Discussion

PCC is a Siddha formulation prepared from purified mercury, sulphur, and gold having potent therapeutic efficacy and rejuvenating activity. Siddha practitioners claim that besides having highly toxic metals in PCC formulation, the techniques involved during the manufacturing procedure such as repeated purification of raw materials and unique preparation methods prescribed in Siddha literature ensure the safety of the drug. To evaluate the safety profile of PCC, acute and repeated oral toxicity studies were performed. Acute oral toxicity study of a test substance forms the crucial pace in the description of any chemicals in the drug development process. Different test methods have been devised by OECD for acute toxicity evaluation. Pũrṇa Cantirotaya Centũram was tested on acute oral exposure as per OECD guideline 423 stepwise dose procedure that utilizes minimal animal usage to determine the toxicity of the test material. Up to the dose level of 2000 mg/kg of body weight, all animals were free of intoxication signs throughout the period of study. Gross pathology examination of animals sacrificed at the end of the study revealed no abnormalities. Thus, the LD_50_ cut-off value was found to be between 2000 and 5000 mg/kg body weight with reference to the Globally Harmonized System of Classification and Labelling of Chemicals [19]. So, the test drug PCC can be classified as Category 5 with low acute toxicity hazard.

Acute toxicity data are of limited clinical application since cumulative toxic effects do occur even at very low doses. Hence, multiple dose studies are usually helpful in evaluating the safety profile of phytomedicines. 28-day repeated oral toxicity study was therefore carried out. Body weight changes are an indicator of adverse side effects, as the animals that survive cannot lose more than 10% of the initial body weight [22]. There were no significant changes in body weight between vehicle control and test groups in both acute and repeated oral drug treatment. Repeated dose toxicity studies were conducted to evaluate the adverse effects of test drug PCC and were carried out to provide information about the possible health hazards likely to arise from repeated exposure over a relatively limited period of time, the possibilities of cumulative effects, and an estimate of the dose at which there is no observed adverse effect. Metals and minerals that are transformed into drug must have excellent therapeutic efficacy and must be safe. It is essential to evaluate the margin of safety between the dose level that produces the therapeutic effect and that which produces the adverse effects. That is to provide benefit to risk assessment [23]. Determination of food consumption was important in the study of safety of a product with therapeutic purpose as proper intake of nutrients is essential to the physiological status of the animals and to the accomplishment of the proper response to the drug tested instead of a false response due to improper nutritional conditions [24]. In water and food consumption, no significant changes were observed in PCC treated groups and this reveals that it did not adversely affect the basic metabolic processes of the experimental animals.

Clinical biochemistry and hematological data hold significant role in determining the toxicity induced by drugs. Blood parameters analysis is relevant to risk evaluation as the haematological system has a higher predictive value for toxicity in humans (91%) when assays involve rodents and nonrodents [25]. Blood forms the main medium of transport for many drugs and xenobiotics in the body and for that matter components of the blood such as red blood cells, white blood cells, haemoglobin, and platelets are at least initially exposed to significant concentrations of toxic compounds. Damage to and destruction of the blood cells are inimical to normal functioning of the body [26]. There is no significant alteration in haematological parameters which indicate that PCC did not affect blood cells production. There is nonsignificant dose dependent increase in the white blood cell count, red blood cell count, and haemoglobin concentration with the escalation of dose (Table 1). The heavy metals lead, mercury, cadmium, and arsenic on subcellular organelle systems following* in vivo* administration have been found to interfere with normal cellular replication and genetic processes [27]. The various blood cells (erythrocytes, leucocytes, and platelets) produced at a turnover rate of about 1 to 3 million per second in a healthy human adult and this value could be altered in certain physiological or pathological states [28]. Certain drugs including alkylating cytotoxic agents affect blood formation rate and the normal range of hematological parameters [29]. There is no haematological variation noted between vehicle control and test drug treated groups. Bilirubin is formed by the breakdown of hemoglobin in the liver, spleen, and bone marrow. An increase in tissue or serum bilirubin concentrations occurs as a result of increased breakdown of RBC (haemolysis) or liver damage, for example, hepatitis or bile duct obstruction. The normal levels of serum bilirubin concentrations at all doses of the PCC used in this study are indicative of nonadverse effects of the test drug on haemoglobin metabolism pathways. The results of the hematological parameters of the present study did not show any worrisome results since all the changes were within the normal expected range for the rat species used in this study.

In preclinical toxicity studies, renal changes are particularly liable to occur because of the high doses given and the fact that the kidneys eliminate many drugs and their metabolites [30]. Creatinine, on the other hand, is mostly derived from endogenous sources by tissue creatinine breakdown. The plasma creatinine concentrations in normal individuals are usually affected by a number of factors such as the muscle mass, high protein diet, and catabolic state; thus serum urea concentration is often considered the more reliable renal function predictor [31]. In the present study there were some significant changes observed in urea, creatinine, cholesterol, and albumin parameters between vehicle control group and low dose 2.5 mg/kg body weight group, which might be due to incidental. Histopathological findings were also normal in low dose group and unsubstantiated these biochemical alterations. In the present study there were no significant changes in the levels of creatinine, urea sodium, potassium and chloride in the sera of other two groups of rats treated with different doses of PCC and therefore they were considered nonnephrotoxic.

Transaminases (SGOT and SGPT) are good indicators of liver function and biomarkers to predict the possible toxicity of drugs [32]. There were no changes in the SGPT and SGOT levels which reveal that PCC did not affect liver function. There were no significant differences in other biochemical parameters between vehicle control and test groups.

The evaluation of histopathological changes in organs remains a cornerstone in safety assessment of medicines [33]. Absolute terminal organ weight and percent relative organ weight indicative of test compound caused changes in functioning of target organs, changes in phospholipid metabolism, over- or undersecretion of enzymes and hormones, hypo/hyperplasia, and possible tissue necrosis [34]. There was no significant difference in absolute organ weights of treated group as compared to vehicle control group. High dose (50 mg/kg b.w.) group exhibits mild hyperplasia in nonglandular area of stomach, in which the dose was with 20 times higher concentration than the therapeutic dose of the test drug. This may be due to stress induced because of repeated drug administration. No abnormality was recorded with respect to gross or histopathological examinations of all other organs examined. There were no signs of toxicity with respect to hematology, serum chemistry, organ weight, and gross and histopathological examinations noted in PCC treated group. Since there were no signs of toxicity with respect to hematology, clinical chemistry, organ weight, and gross and histopathological examinations noted in PCC satellite group, it can be inferred that the test drug PCC will not produce delayed onset of toxicity.

## 5. Conclusion

Based on these results, drug doses lower than or equal to 50 mg/kg b.w. in rats did not show any biochemical, histological, or haematological signs of toxicity. The human equivalent dose for high dose given in rat is 480 mg based on body surface area conversion factor [35], which indicates that the traditionally recommended dose (65–130 mg for human adult) could be very safe.

## Figures and Tables

**Table 1:** Effect of Pũrṇa Cantirotaya Centũram on hematological parameters.

Parameters	Control (honey)	Low dose (2.5 mg/kg b.w.)	Mid dose (25 mg/kg b.w.)	High dose (50 mg/kg b.w.)
WBC (×10^9^/L)	8.06 ± 5.06	5.26 ± 4.11	6.87 ± 4.68	7.48 ± 6.19
Lymph [%]	62.13 ± 11.72	61.89 ± 20.71	56.05 ± 7.99	60.22 ± 5.43
Mon [%]	4.30 ± 1.79	3.42 ± 1.38	3.66 ± 0.35	4.46 ± 1.52
Gran [%]	34.32 ± 9.79	34.69 ± 19.48	40.29 ± 7.99	35.32 ± 3.97
RBC (×10^12^/L)	7.16 ± 3.58	5.29 ± 3.57	6.70 ± 3.06	7.76 ± 3.66
HGB (g/dL)	12.11 ± 6.03	8.90 ± 6.09	11.27 ± 5.77	12.45 ± 5.84
HCT [%]	35.02 ± 18.06	26.36 ± 18.17	31.00 ± 14.24	37.33 ± 17.54
MCV (fL)	51.66 ± 5.99	49.97 ± 3.42	48.52 ± 2.69	49.07 ± 1.64
MCH (pg)	17.82 ± 2.90	16.68 ± 1.26	16.38 ± 1.05	16.27 ± 0.58
MCHC (g/dL)	34.67 ± 2.63	33.55 ± 1.57	30.57 ± 10.74	33.27 ± 0.57
RDW [%]	13.05 ± 3.81	11.44 ± 0.89	11.650 ± 1.23	11.43 ± 0.94
PLT (×10^9^/L)	200.70 ± 78.19	210.9 ± 55.6	227.5 ± 55.95	196.50 ± 144.17
MPV (fL)	6.53 ± 0.62	6.76 ± 1.03	6.38 ± 0.76	6.50 ± 0.64
PDW	15.30 ± 0.47	15.74 ± 0.89	15.45 ± 0.55	15.130 ± 0.46
PCT [%]	0.131 ± 0.04	0.130 ± 0.03	0.142 ± 0.035	0.130 ± 0.10

Comparison was made between vehicle control and test groups using the one-way ANOVA test followed by Dunnett's test. Values are mean ± SD for 10 rats in each group (*P* ≤ 0.05).

RBC: red blood corpuscles; HGB: hemoglobin; HCT: hematocrit; MCV: mean corpuscular volume; MCH: mean corpuscular hemoglobin; MCHC: mean corpuscular hemoglobin concentration; WBC: white blood cells; PLT: platelets; MPV: mean platelet volume; PCT: plateletcrit; RDW: red blood cell distribution width; PDW: platelet distribution width.

**Table 2:** Effect of Pũrṇa Cantirotaya Centũram on biochemistry parameters.

Parameters	Control (honey)	Low dose (2.5 mg/kg b.w.)	Mid dose (25 mg/kg b.w.)	High dose (50 mg/kg b.w.)
Glucose (mg/dL)	111.20 ± 21.07	90.20 ± 27.93	94.20 ± 29.65	102.10 ± 38.30
Blood urea (mg/dL)	34.90 ± 8.97	46.60 ± 9.14^*^	39.90 ± 8.99	29.80 ± 6.4
Creatinine (mg%)	0.73 ± 0.24	0.990 ± 0.36^*^	0.92 ± 0.36	0.62 ± 0.12
Cholesterol (mg/dL)	112.70 ± 38.86	153.30 ± 45.32^*^	110.70 ± 42.06	94.80 ± 17.88
TGL (mg/dL)	89.00 ± 18.08	87.10 ± 29.77	75.80 ± 19.11	87.40 ± 25.20
Total protein (g%)	8.32 ± 0.56	8.38 ± 0.64	10.24 ± 2.17	10.15 ± 3.67
Albumin (g%)	2.79 ± 0.35	3.04 ± 0.42^*^	2.35 ± 0.45	2.98 ± 0.43
S. bilirubin (mg/dL)	0.710 ± 0.119	0.74 ± 0.06	0.630 ± 0.14	0.610 ± 0.21
SGOT (IU/L)	168.00 ± 14.81	165.50 ± 19.092	167.80 ± 16.19	169.10 ± 12.76
SGPT (IU/L)	62.00 ± 8.91	57.70 ± 14.855	49.40 ± 8.05	67.60 ± 41.21
Na^+^ (mEq/L)	130.10 ± 5.56	132.20 ± 2.300	133.10 ± 1.66	133.10 ± 2.02
K^+^ (mEq/L)	5.76 ± 2.43	6.26 ± 1.1909	5.590 ± 1.20	5.240 ± 0.72
Cl^−^ (mEq/L)	107.00 ± 1.41	106.30 ± 1.947	105.90 ± 1.79	105.90 ± 1.79

Comparison was made between vehicle control and test groups using the one-way ANOVA test followed by Dunnett's test. Values are mean ± SD for 10 rats in each group (*P* ^*^ < 0.05).

TGL: triglycerides, SGOT: serum glutamic oxaloacetic transaminase, SGPT: serum glutamic pyruvic transaminase, Na^+^: sodium, K^+^: potassium, and Cl^−^: chloride.

**Table 3:** Effect of Pũrṇa Cantirotaya Centũram on relative organ weight (in grams).

Organs	Control (honey)	Low dose (2.5 mg/kg b.w.)	Mid dose (25 mg/kg b.w.)	High dose (50 mg/kg b.w.)
Brain	0.938 ± 0.136	1.028 ± 0.212	0.939 ± 0.105	0.865 ± 0.178
Heart	0.411 ± 0.062	0.419 ± 0.116	0.404 ± 0.113	0.436 ± 0.098
Lung	0.917 ± 0.473	0.882 ± 0.347	1.027 ± 0.604	0.69 ± 0.103
Liver	3.875 ± 0.560	4.107 ± 0.814	3.799 ± 0.863	3.986 ± 1.33
Stomach	0.507 ± 0.095	0.530 ± 0.101	0.539 ± 0.133	0.527 ± 0.130
Spleen	0.9 ± 0.114	0.833 ± 0.190	0.816 ± 0.104	0.975 ± 0.536
Thymus	0.402 ± 0.084	0.392 ± 0.131	0.381 ± 0.087	0.386 ± 0.072
Kidney right	0.151 ± 0.034	0.185 ± 0.049	0.15 ± 0.059	0.149 ± 0.023
Kidney left	0.528 ± 0.103	0.55 ± 0.099	0.547 ± 0.134	0.523 ± 0.150
Testis	1.39 ± 0.219	1.67 ± 0.376	1.55 ± 0.112	1.49 ± 0.258
Ovary	0.072 ± 0.019	0.057 ± 0.026	0.058 ± 0.023	0.075 ± 0.023

Comparison was made between vehicle control and test groups using the one-way ANOVA test followed by Dunnett's test. Values are mean ± SD for 10 rats in each group (*P* < 0.05).

## References

[1] Patwardhan B., Hooper M. J. (1992). Ayurveda and future drug development. *Journal of Alternative and Complementary Medicine*.

[2] Subbarayappa B. V. (1997). Siddha medicine: an overview. *The Lancet*.

[3] Utthamarayan K. S. (2003). *HPIM. Siddha Maruthuvanga Surukkam*.

[4] (2008). *Tamil maruthuva nool varisai enn-8, Sarakku SutthiSei Muraigal*.

[5] Thiagarajan R. (2004). *Gunapaadam-Thathu Jeeva Vaguppu*.

[6] Shah Z. A., Vohora S. B. (2002). Antioxidant/restorative effects of calcined gold preparations used in Indian systems of medicine against global and focal models of ischaemia. *Pharmacology and Toxicology*.

[7] Chandra D., Mandal A. K. (2000). Toxicological and pharmacological study of Navbal Rasayan—a metal based formulation. *Indian Journal of Pharmacology*.

[8] Lipsky P. E. (1984). Remittive therapy in rheumatoid arthritis: clinical uses and mechanisms of action. *Agents and Actions*.

[9] Singh N., Anand C. (2012). Swarna Bhasma and gold compounds: an innovation of pharmaceutics for illumination of therapeutics. *International Journal of Research in Ayurveda and Pharmacy*.

[10] Paul W., Sharma C. P. (2011). Blood compatibility studies of *Swarna bhasma* (gold *bhasma*), an *Ayurvedic* drug. *International Journal of Ayurveda Research*.

[11] Anita K., Anita S., Venketeswarlu U., Kumar G. V. (2013). Toxicological evaluation of *Rasa-Sindoor* in Albino rats. *International Ayurvedic Medical Journal*.

[12] Tharakan S. T., Kuttan G., Kuttan R., Kesavan M., Austin S., Rajagopalan K. (2010). Toxicity studies of Sidha medicine—Rasagandhi Mezhugu. *The Open Toxicology Journal*.

[13] Kroes R., Walker R. (2004). Safety issues of botanicals and botanical preparations in functional foods. *Toxicology*.

[14] Vohora S. B., Dobrowolski J. W. (1990). *New Horizons of Health Aspects of Elements*.

[15] Abdulla M., Vohora S. B., Athar M. (1995). *Trace and Toxic Elements in Nutrition and Health*.

[16] WHO (1996). *Trace Elements in Human Nutrition and Health*.

[17] Kuppuswami Mudaliyar K. N., Uthamarayan Su K. (1998). *Siddha Vaidya Thirattu*.

[18] Thiagarajan S. P. (1998). *Sirappu Maruthuvam*.

[19] OECD (2001). *OECD Guideline for Testing of Chemicals, Acute Oral Toxicity-Acute Toxic Class Method: TG 423-Adopted*.

[20] OECD (1995). *OECD Guideline for Testing of Chemicals, Repeated Dose 28-day Oral Toxicity Study in Rodents: TG 407-Adopted*.

[21] Gulkarni S. K. (1999). *Hand Book of Experimental Pharmacology*.

[22] Wonder H. K. M., George K. A., Gyasi E. B. (2011). Acute and sub-acute toxicity studies of the ethanolic extract of the aerial parts of *Hilleria latifolia* (Lam.) H. Walt. (Phytolaccaceae) in rodents. *West African Journal of Pharmacy*.

[23] Prajapathi P. K., Sarkar P. K., Nayak S. V., Joshi R. D., Ravishankar B. (2006). Safety and toxicity profile of some metallic preparations of Ayurveda. *Ancient Science of Life*.

[24] Sathish R., Anbu J., Murgesan M., Anjana A., Kumar A. (2012). Toxicity study on siddha formulation mega Sanjeevi Mathirai in albino rats. *International Journal of Pharma and Bio Sciences*.

[25] Olson H., Betton G., Robinson D. (2000). Concordance of the toxicity of pharmaceuticals in humans and in animals. *Regulatory Toxicology and Pharmacology*.

[26] Adeneye A. A., Ajagbonna O. P., Adeleke T. I., Bello S. O. (2006). Preliminary toxicity and phytochemical studies of the stem bark aqueous extract of Musanga cecropioides in rats. *Journal of Ethnopharmacology*.

[27] Jesse B. (1982). *Animal Anatomy and Physiology*.

[28] Guyton A. C., Hall J. E. (2000). *Textbook of Medical Physiology*.

[29] Zuk A., Targosz-Korecka M., Szymonski M. (2011). Effect of selected drugs used in asthma treatment on morphology and elastic properties of red blood cells. *International Journal of Nanomedicine*.

[30] El Hilaly J., Israili Z. H., Lyoussi B. (2004). Acute and chronic toxicological studies of *Ajuga iva* in experimental animals. *Journal of Ethnopharmacology*.

[31] Shatoor A. S. (2011). Acute and sub-acute toxicity of Crataegus aronia syn. Azarolus (L.) whole plant aqueous extract in wistar rats. *The American Journal of Pharmacology and Toxicology*.

[32] Mayne P., Mayne P. D. (1994). *Clinical Chemistry in Diagnosis and Treatment*.

[33] Greaves P. (2007). *Histopathology of Preclinical Toxicity Studies: Interpretation and Relevance in Drug Safety Evaluation*.

[34] Dhumal R., Patil P., Selkar N., Chawda M., Vahlia M., Vanage G. (2013). Sub-chronic safety evaluation of ayurvedic immunostimulant formulation “immuforte” in rats in reverse pharmacology. *Toxicology International*.

[35] Freireich E. J., Gehan E. A., Rall D. P., Schmidt L. H., Skipper H. E. (1966). Quantitative comparison of toxicity of anticancer agents in mouse, rat, hamster, dog, monkey, and man. *Cancer Chemotherapy Reports*.

